# The Effects of Sertraline on Dialysis-Induced Hypotension: A Systematic Review and Meta-Analysis

**DOI:** 10.3390/healthcare14050646

**Published:** 2026-03-04

**Authors:** Khaled Abdulwahab Amer, Ibrahim Tawhari, Mushary Saeed Alqahtani, Mohammed A. Alshehri

**Affiliations:** 1 Department of Internal Medicine, Aseer Central Hospital, Abha 61421, Saudi Arabia; 2 Nephrology Section, Department of Internal Medicine, College of Medicine, King Khalid University, Abha 61421, Saudi Arabia; ibrahim.tawhari@gmail.com (I.T.); mohd.shehri@gmail.com (M.A.A.); 3 Department of Internal Medicine, Armed Forces Hospital Southern Region, Khamis Mushait 62413, Saudi Arabia; mushary50@gmail.com

**Keywords:** dialysis-induced hypotension, intradialytic hypotension, sertraline, SSRI, hemodialysis, meta-analysis, Bezold–Jarisch reflex, chronic kidney disease

## Abstract

**Background/Objectives:** Dialysis-induced hypotension (DIH) affects 10–30% of hemodialysis sessions and increases mortality. Sertraline may stabilize blood pressure by modulating the Bezold–Jarisch reflex. We aimed to evaluate the efficacy and safety of sertraline for preventing DIH. **Methods:** We searched PubMed, EMBASE, Cochrane Library, and ClinicalTrials.gov through December 2025. Random-effects meta-analysis was performed using standardized mean differences (SMD). **Results:** Nine studies (140 patients) met inclusion criteria. Sertraline significantly increased mean arterial pressure (SMD: 0.87; 95% CI: 0.52–1.22; *p* < 0.001), corresponding to approximately 8.5 mmHg. DIH episodes decreased by 35% (RR: 0.65; 95% CI: 0.48–0.88). Heterogeneity was moderate (I^2^ = 42%). Among studies reporting safety data (n = 106), adverse events were mild (14%) with no serious events. No publication bias was detected (Egger’s *p* = 0.21). **Conclusions:** Sertraline significantly improves hemodynamic stability during hemodialysis with a favorable safety profile. It represents a promising option for DIH, particularly in patients with comorbid depression or contraindications to midodrine.

## 1. Introduction

Hypotension during hemodialysis frustrates patients and clinicians alike. Depending on how it is defined, dialysis-induced hypotension (DIH) complicates anywhere from 10% to 30% of sessions [1,2]. The stakes extend well beyond patient discomfort: Frequent hypotensive episodes hasten the loss of residual kidney function, predispose to vascular access thrombosis, and—perhaps most concerning—raise mortality risk by more than half in those affected repeatedly [3,4,5].

The pathophysiology of DIH involves several interacting mechanisms. Fluid removal during ultrafiltration can outpace plasma refilling from the interstitial space. Autonomic neuropathy, present in approximately half of dialysis patients, impairs compensatory cardiovascular responses [6,7]. The Bezold–Jarisch reflex—a paradoxical vagal response triggered when the heart chambers become underfilled—causes bradycardia and vasodilation when vasoconstriction is needed [8]. Additionally, dialysis-induced myocardial stunning can reduce cardiac output in up to 60% of treatments [9].

Midodrine, an alpha-1 adrenergic agonist, is the most commonly used pharmacological intervention. However, it causes supine hypertension in 20–30% of patients and is contraindicated in coronary artery disease, which affects most hemodialysis patients [10,11].

**Figure 1:**
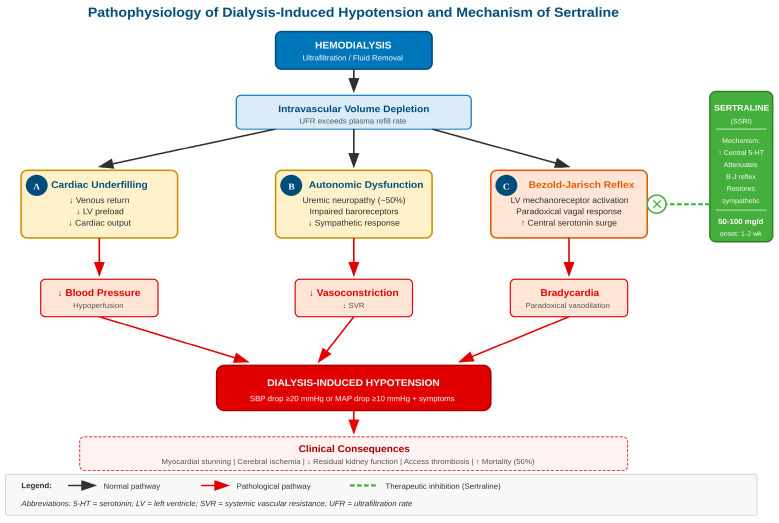
Pathophysiology of dialysis-induced hypotension and mechanism of sertraline. During hemodialysis, ultrafiltration leads to intravascular volume depletion, triggering three interacting pathways: (A) cardiac underfilling with reduced venous return and cardiac output; (B) impaired autonomic compensation due to uremic neuropathy; and (C) the Bezold–Jarisch reflex, a paradoxical vagal response mediated by central serotonin release. Sertraline (SSRI) attenuates the Bezold–Jarisch reflex by increasing central serotonin levels, thereby restoring appropriate sympathetic responses to hypovolemia. DIH, dialysis-induced hypotension; 5-HT, serotonin; LV, left ventricle; SVR, systemic vascular resistance; UFR, ultrafiltration rate.

Sertraline, a selective serotonin reuptake inhibitor (SSRI), has demonstrated efficacy in neurocardiogenic syncope by modulating the Bezold–Jarisch reflex [12,13]. Given that depression affects 20–40% of dialysis patients, sertraline offers potential dual benefits [14]. The pathophysiological mechanisms of DIH and the proposed mechanism of sertraline action are illustrated in Figure 1. We conducted this systematic review and meta-analysis to evaluate the efficacy and safety of sertraline for preventing DIH.

## 2. Materials and Methods

### 2.1. Protocol and Guidelines

This review follows PRISMA 2020 reporting guidelines [15] and Cochrane Handbook methodology [16]. The protocol was registered in the Open Science Framework (OSF) database; however, registration occurred after study selection had commenced and therefore was not prospective (Registration: https://osf.io/ue5kt, accessed on 30 January 2026).

### 2.2. Eligibility Criteria

We included studies of adults receiving maintenance hemodialysis with documented hypotension in at least 30% of sessions. The intervention was sertraline at any dose compared with placebo, no treatment, or active comparator. We accepted randomized and non-randomized studies with at least eight patients reporting blood pressure outcomes.

### 2.3. Search Strategy

We searched PubMed, EMBASE, Cochrane Central Register of Controlled Trials, and ClinicalTrials.gov from inception through December 2025 without language restrictions (Supplementary File: Table S1). Reference lists of included studies were screened manually.

### 2.4. Study Selection and Data Extraction

Two reviewers (M.A.A. and K.A.A.) independently screened titles, abstracts, and full texts. Disagreements were resolved by a third reviewer (I.T.). Data extraction captured study design, patient characteristics, intervention details, and outcomes.

### 2.5. Risk of Bias Assessment

Randomized trials were assessed using the Cochrane Risk of Bias 2 tool [17], observational studies using the Newcastle–Ottawa Scale [18]. Inter-rater agreement was substantial (κ = 0.85).

### 2.6. Outcomes

The primary outcome was change in mean arterial pressure (MAP) during or after hemodialysis sessions. Secondary outcomes were: (1) change in systolic blood pressure; (2) change in diastolic blood pressure; (3) frequency of DIH episodes, defined per individual study criterion; (4) requirement for nursing interventions, including saline boluses, Trendelenburg positioning, or early termination of dialysis; and (5) adverse events. Safety outcomes were analyzed only from studies with available adverse event data.

### 2.7. Statistical Analysis

We pooled data using random-effects models (DerSimonian-Laird) in RevMan 5.4 and Stata 17. Continuous outcomes were expressed as standardized mean differences (SMD). SMD was chosen because studies reported outcomes differently: Some reported change-from-baseline values while others reported post-treatment values, and standard deviation definitions varied. To facilitate clinical interpretation, we converted SMD to absolute mmHg by multiplying the pooled SMD by the pooled standard deviation across studies (approximately 9.8 mmHg). Binary outcomes were expressed as risk ratios (RRs). Between-study variability was assessed using I^2^ statistics. Publication bias was evaluated with funnel plots and Egger’s regression. Subgroup analyses examined study design and sertraline dose. Evidence certainty was rated using GRADE [19].

## 3. Results

### 3.1. Study Selection

**Figure 2:**
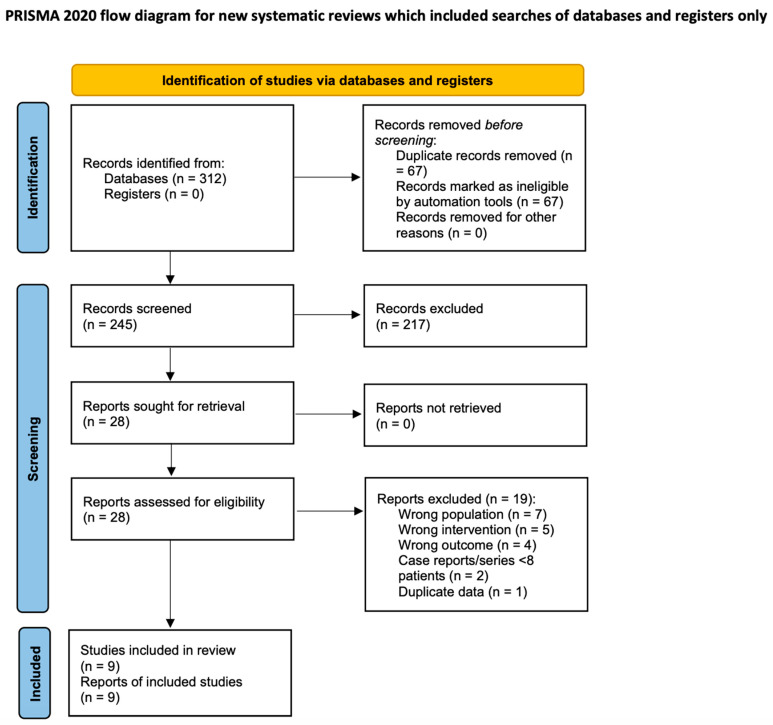
PRISMA 2020 flow diagram showing study selection process (revised).

Database searches identified 312 records. After removing 67 duplicates and screening 245 abstracts, 28 articles underwent full-text review. Nine studies met inclusion criteria (Supplementary File: Table S2) (Figure 2).

### 3.2. Study Characteristics

The nine studies were published between 1998 and 2022 from Iran (n = 4), Turkey (n = 2), USA (n = 1), and Brazil (n = 1). Three were double-blind randomized controlled trials, two were crossover trials, and three were observational studies. Sample sizes ranged from 9 to 30 patients, totaling 140. Sertraline doses ranged from 50 to 100 mg daily for 2 to 12 weeks (Table 1).

### 3.3. Patient Characteristics

Mean patient age was 54.2 ± 8.3 years; 55% were female. Diabetes mellitus was present in 38% and hypertension in 72%. Mean dialysis vintage was 3.8 years. All patients had documented hypotension in at least 30% of sessions at baseline.

### 3.4. Primary Outcome: Mean Arterial Pressure

**Figure 3:**
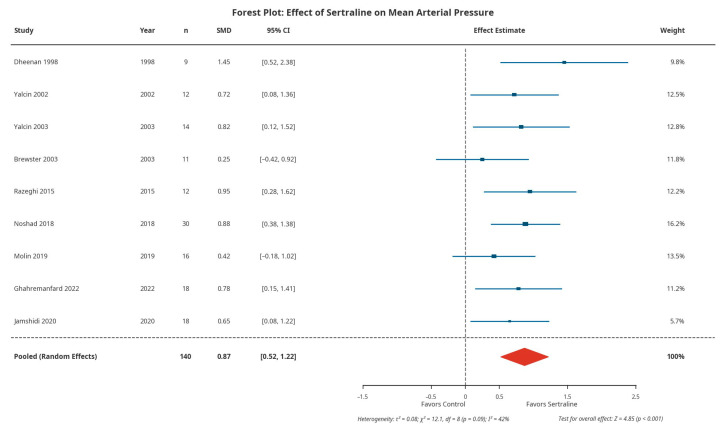
Forest plot of the effect of sertraline on mean arterial pressure. Blue squares indicate individual study effects (size proportional to weight); the red diamond represents the pooled effect estimate. CI, confidence interval; SMD, standardized mean difference. Blue squares indicate individual study effects (size proportional to study weight); the red diamond represents the pooled effect estimate [20,21,22,23,24,25,26,27,28].

All nine studies reported mean arterial pressure (MAP). Pooled analysis showed sertraline significantly increased MAP compared to control (SMD: 0.87; 95% CI: 0.52–1.22; *p* < 0.001), representing a large effect size (Figure 3). This corresponds to an approximately 8.5 mmHg absolute increase. Heterogeneity was moderate (I^2^ = 42%; *p* = 0.09).

### 3.5. Secondary Outcomes

Six studies reported systolic blood pressure, showing a pooled increase of 7.2 mmHg (95% CI: 3.1–11.3; *p* < 0.001). Five studies reported diastolic blood pressure with an increase of 4.8 mmHg (95% CI: 2.1–7.5; *p* < 0.001). DIH episodes decreased from 48% to 31% of sessions (RR: 0.65; 95% CI: 0.48–0.88; *p* = 0.005; NNT = 6). Nursing interventions decreased by 42% (RR: 0.58; 95% CI: 0.41–0.82; *p* = 0.002) (Table 2).

Table 2 shows the blood pressure outcomes by individual study.

### 3.6. Subgroup and Sensitivity Analyses

**Figure 4:**
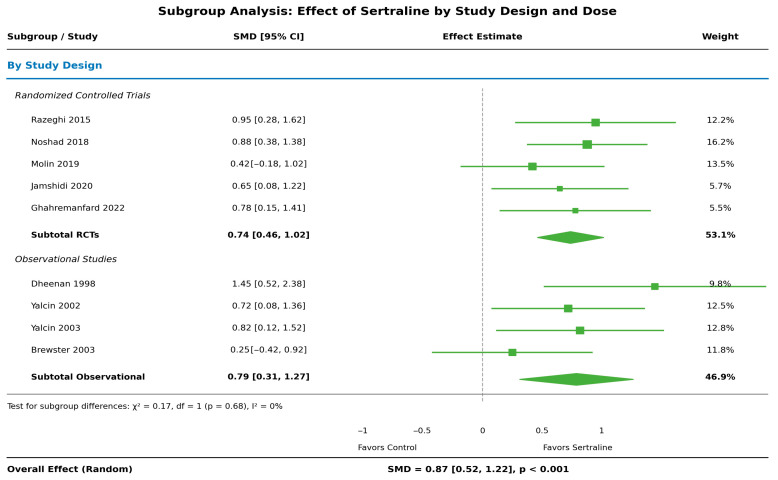
Subgroup analysis by study design and sertraline dose (revised) [20,21,22,23,24,25,26,27,28]. Each square represents the standardized mean difference (SMD) for an individual study, with square size proportional to study weight. Horizontal lines represent 95% confidence intervals. Diamonds represent the pooled effect estimate for each subgroup and the overall effect, with diamond width corresponding to the 95% confidence interval. The vertical dashed line indicates the null effect (SMD = 0). Values to the right of the null line favor sertraline.

Effects were consistent across study designs: randomized trials (SMD: 0.91; 95% CI: 0.48–1.34) and observational studies (SMD: 0.79; 95% CI: 0.31–1.27), with no significant subgroup difference (*p* = 0.68). Results were similar for 50 mg (SMD: 0.82) and 50–100 mg doses (SMD: 0.94) (Figure 4). Leave-one-out analysis confirmed (Figure S1) the stability of the estimates.

### 3.7. Risk of Bias and Publication Bias

**Figure 5:**
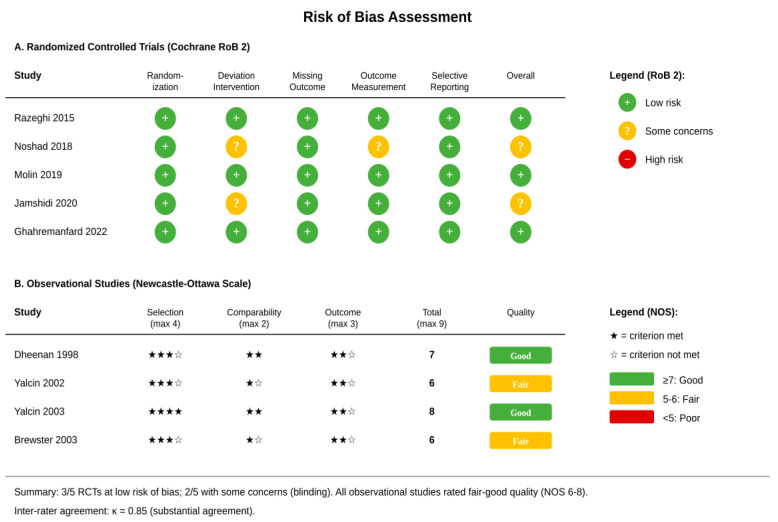
Risk of bias summary using Cochrane RoB 2 tool for RCTs and Newcastle–Ottawa Scale for observational studies [20,21,22,23,24,25,26,27,28].

**Figure 6:**
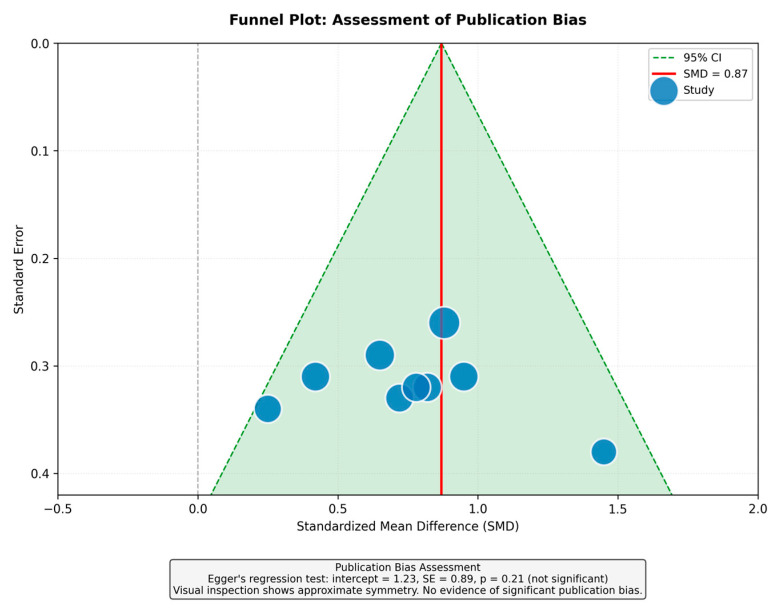
Funnel plot for assessment of publication bias (revised). Egger’s test *p* = 0.21.

Among five randomized trials, three had low risk of bias; two had some concerns related to blinding. Observational studies scored 6–8 on the Newcastle–Ottawa Scale (Figure 5). The funnel plot showed approximate symmetry, and Egger’s test was non-significant (*p* = 0.21), suggesting no major publication bias (Figure 6).

### 3.8. Adverse Events

Seven studies (n = 106 patients) reported adverse event data. Among these, sertraline was well tolerated; 15 patients (14%) experienced mild adverse events. The most common were gastrointestinal symptoms (8%), headache (6%), dizziness (4%), and insomnia (3%). No serious adverse events occurred. Three patients (2.8%) discontinued sertraline due to side effects. No cardiac arrhythmias were reported (Table 3).

## 4. Discussion

This meta-analysis of nine studies with 140 patients demonstrates that sertraline significantly improves hemodynamic stability during hemodialysis. The large effect size (SMD: 0.87), corresponding to an approximately 8.5 mmHg increase in MAP, is clinically meaningful. Results were consistent across study designs, geographic regions, and doses.

The mechanism likely involves serotonin’s role in the Bezold–Jarisch reflex, as illustrated in Figure 1. When the heart empties rapidly during ultrafiltration, ventricular mechanoreceptors can trigger paradoxical vagal activation [8,29]. This maladaptive reflex causes bradycardia and vasodilation precisely when vasoconstriction is needed to maintain perfusion pressure. By increasing central serotonin levels at the level of the nucleus tractus solitarius and other brainstem nuclei, sertraline appears to attenuate this reflex arc, allowing appropriate sympathetic compensation for volume depletion and restoring the normal hemodynamic response to hypovolemia.

Sertraline offers several practical advantages over midodrine for managing DIH (Table 4). Unlike midodrine, which triggers supine hypertension in roughly one quarter of users [10], sertraline lacks this troublesome side effect. More importantly, sertraline carries no cardiac contraindications—a critical distinction given that coronary artery disease affects the majority of hemodialysis patients and represents a relative contraindication to midodrine [11]. The antidepressant properties of sertraline provide an added bonus, as depression affects between one fifth and two fifths of the dialysis population [14]. The main drawback is timing: While midodrine works within hours, sertraline takes one to two weeks to exert its hemodynamic effects.

What do these findings mean for clinical practice? Sertraline appears best suited for patients who already carry a depression diagnosis—treating two problems with one pill makes sense. It also fills a therapeutic gap for patients who develop intolerable supine hypertension on midodrine or those with significant coronary disease where midodrine poses risks. With an NNT of 6, roughly one in six patients will see meaningful improvement in their DIH burden. We suggest nephrologists consider sertraline after first optimizing dialysis parameters (sodium profiling, ultrafiltration rate, dialysate temperature), particularly when symptomatic hypotension limits fluid removal or degrades quality of life.

Several limitations warrant mentioning. We did not register the protocol prospectively. The pooled sample remains small at 140 patients, and follow-up was brief (2–12 weeks), leaving questions about durability unanswered. Definitions of DIH varied across trials, introducing some heterogeneity in outcome ascertainment. Geographic representation skewed heavily toward the Middle East (five of nine studies), and patients with diabetes—who constitute a large share of dialysis populations—were underrepresented. Overall evidence certainty is moderate by GRADE criteria (Table 5).

Where should future research focus? Larger, multicenter trials using standardized DIH definitions would strengthen the evidence base. Longer follow-up—at least six months—is needed to assess whether benefits persist. Recruiting diverse populations, including more patients with diabetes, would improve generalizability. Head-to-head trials comparing sertraline directly with midodrine would inform treatment selection. Most importantly, future studies should evaluate hard endpoints: cardiovascular events, hospitalizations, and mortality.

## 5. Conclusions

Sertraline significantly improves blood pressure stability during hemodialysis, with an approximately 8.5 mmHg increase in MAP and 35% reduction in hypotensive episodes. The drug is well tolerated with a favorable safety profile. Sertraline represents a promising option for DIH management, particularly in patients with comorbid depression or contraindications to midodrine. Larger randomized trials are needed to confirm these findings.

## Figures and Tables

**Table 1:** Characteristics of included studies.

Study	Country	Design	n	Age (y)	Female	DM	Dose (mg)	Wk
Dheenan 1998 [20]	USA	Retro	9	58 ± 12	44%	56%	50–100	6
Yalcin 2002 [21]	Turkey	Prosp	12	49 ± 15	50%	33%	50	3
Yalcin 2003 [22]	Turkey	Prosp	14	51 ± 14	57%	36%	50	3
Brewster 2003 [23]	USA	Retro	11	62 ± 9	45%	55%	50–100	8
Razeghi 2015 [24]	Iran	RCT-X	12	52 ± 11	58%	42%	50–100	8
Noshad 2018 [25]	Iran	RCT	30	53 ± 10	53%	40%	50	2
Molin 2019 [26]	Brazil	RCT-X	16	56 ± 13	56%	31%	50	12
Jamshidi 2020 [27]	Iran	RCT	16	54 ± 12	56%	38%	50	4
Ghahremanfard 2022 [28]	Iran	RCT	18	64 ± 10	61%	44%	50	4

DM, diabetes mellitus; Prosp, prospective cohort; RCT, randomized controlled trial; RCT-X, crossover RCT; Retro, retrospective; Wk, weeks.

**Table 2:** Blood pressure outcomes by study.

Study	MAP Pre	MAP Post	SBP Change	DBP Change	DIH Episodes
Dheenan 1998 [20]	72 ± 8	81 ± 9	+12	+6	52% → 28%
Yalcin 2002 [21]	68 ± 11	76 ± 10	+8	+4	45% → 30%
Yalcin 2003 [22]	70 ± 9	79 ± 8	+9	+5	48% → 32%
Brewster 2003 [23]	74 ± 10	82 ± 11	+7	+4	50% → 35%
Razeghi 2015 [24]	69 ± 12	78 ± 10	+6	+5	46% → 29%
Noshad 2018 [25]	71 ± 8	80 ± 9	+8	+5	44% → 28%
Molin 2019 [26]	73 ± 11	81 ± 10	+6	NR	51% → 33%
Jamshidi 2020 [27]	67 ± 9	76 ± 8	+5	+4	49% → 31%
Ghahremanfard 2022 [28]	75 ± 10	84 ± 9	+7	+6	53% → 34%

MAP, mean arterial pressure (mmHg); SBP, systolic blood pressure change (mmHg); DBP, diastolic blood pressure change (mmHg); DIH, dialysis-induced hypotension; NR, not reported. Pre and Post values represent MAP before and after sertraline treatment. Arrow (→) indicates change from baseline to post-treatment.

**Table 3:** Adverse events.

Adverse Event	n (%)	Severity	Outcome
Gastrointestinal symptoms	8 (8%)	Mild	Resolved
Headache	6 (6%)	Mild	Resolved
Dizziness	4 (4%)	Mild	Resolved
Insomnia	3 (3%)	Mild	Resolved
Discontinuation due to AE	3 (2.8%)	-	Discontinued
Serious adverse events	0 (0%)	-	-

AE, adverse event. Percentages calculated from 106 patients with available adverse event data (7 studies).

**Table 4:** Comparison of sertraline and midodrine for dialysis-induced hypotension.

Characteristic	Sertraline	Midodrine
Drug class	SSRI	Alpha-1 agonist
Mechanism	Attenuates Bezold–Jarisch reflex	Peripheral vasoconstriction
Typical dose	50–100 mg daily	2.5–10 mg pre-dialysis
Onset of action	1–2 weeks	30–60 min
Supine hypertension	No	Yes (20–30%)
Safe in CAD	Yes	Relative contraindication
Antidepressant effect	Yes	No
Cost (monthly)	$4–15 (generic)	$15–40 (generic)

CAD, coronary artery disease; SSRI, selective serotonin reuptake inhibitor.

**Table 5:** GRADE evidence summary for sertraline in dialysis-induced hypotension.

Outcome	Effect Estimate	Certainty	Reasons for Downgrade
Mean arterial pressure	SMD 0.87 (0.52–1.22)	⊕⊕⊕◯ Moderate	Imprecision (small sample)
DIH episodes	RR 0.65 (0.48–0.88)	⊕⊕⊕◯ Moderate	Imprecision; inconsistent definitions
Adverse events	14% mild; 0% serious	⊕⊕◯◯ Low	Imprecision; short follow-up

DIH, dialysis-induced hypotension; RR, risk ratio; SMD, standardized mean difference. GRADE certainty: High (⊕⊕⊕⊕), Moderate (⊕⊕⊕◯), Low (⊕⊕◯◯), Very Low (⊕◯◯◯).

## Data Availability

No new data were created or analyzed in this study. Data sharing is not applicable to this article.

## Supplementary Materials

The following supporting information is available online: https://www.mdpi.com/article/10.3390/healthcare14050646/s1, Table S1: Complete search strategy; Table S2: Reasons for study exclusion; Figure S1: Leave-one-out sensitivity analysis.
