# Supplementary material for: The Effects of Sertraline on Dialysis-Induced Hypotension: A Systematic Review and Meta-Analysis
Source: Healthcare (Basel). 2026 Mar 4;14(5):646. doi: 10.3390/healthcare14050646 (PMC12984499; doi:10.3390/healthcare14050646)
Supplement: Supplementary file 1 [file healthcare-14-00646-s001.zip › healthcare-4153764-supplementary.pdf]

Supplementary Materials

The Effects of Sertraline on Dialysis-Induced Hypotension: A Systematic Review and Meta-Analysis

Table S1. Complete Search Strategy

PubMed Search (December 2025):

("sertraline"[MeSH Terms] OR "sertraline"[Title/Abstract] OR "Zoloft"[Title/Abstract]) AND ("hemodialysis"[MeSH Terms] OR "renal dialysis"[MeSH Terms] OR "dialysis"[Title/Abstract] OR "hemodialysis"[Title/Abstract]) AND ("hypotension"[MeSH Terms] OR "hypotension"[Title/Abstract] OR "blood pressure"[Title/Abstract])

EMBASE Search:

('sertraline'/exp OR sertraline:ti,ab OR zoloft:ti,ab) AND ('hemodialysis'/exp OR dialysis:ti,ab OR hemodialysis:ti,ab) AND ('hypotension'/exp OR hypotension:ti,ab OR 'blood pressure':ti,ab)

Cochrane Library Search:

#1 MeSH descriptor: [Sertraline] explode all trees #2 sertraline OR zoloft:ti,ab,kw #3 MeSH descriptor: [Renal Dialysis] explode all trees #4 dialysis OR hemodialysis:ti,ab,kw #5 MeSH descriptor: [Hypotension] explode all trees #6 hypotension OR blood pressure:ti,ab,kw #7 (#1 OR #2) AND (#3 OR #4) AND (#5 OR #6)

Table S2. Reasons for Study Exclusion

**Reason for Exclusion**	**n**
Not dialysis population	7
No blood pressure outcome	5
Case report or case series <8 patients	4
Review article or editorial	3
Duplicate publication	1
**Total excluded**	**20**

Figure S1. Leave-One-Out Sensitivity Analysis

Leave-one-out sensitivity analysis was performed by sequentially removing each study and recalculating the pooled effect estimate. Results demonstrated stability of findings:

**Study Removed**	**SMD (95% CI)**
Dheenan 1998	0.84 (0.48–1.20)
Yalcin 2002	0.89 (0.52–1.26)
Yalcin 2003	0.86 (0.49–1.23)
Brewster 2003	0.88 (0.51–1.25)

Razeghi 2015	0.90 (0.53–1.27)
Noshad 2018	0.82 (0.44–1.20)
Molin 2019	0.85 (0.48–1.22)
Jamshidi 2020	0.91 (0.54–1.28)
Ghahremanfard 2022	0.83 (0.46–1.20)
**Pooled (all studies)**	**0.87 (0.52–1.22)**

SMD, standardized mean difference; CI, confidence interval. The pooled estimate remained significant regardless of which study was removed, confirming the robustness of findings.
